# Evaluating the safety profile of anti-platelet therapy in patients undergoing elective inguinal hernia repair: a systematic review and meta-analysis

**DOI:** 10.1007/s11845-023-03480-w

**Published:** 2023-08-01

**Authors:** Matthew G. Davey, William P. Joyce

**Affiliations:** 1 Royal College of Surgeons Ireland, 123 St. Stephens Green, Dublin 2, D02 YN77 Ireland; 2 Department of Surgery, Galway Clinic, Co., Galway, H91 HHT0 Ireland

**Keywords:** Anti-platelet therapy, Aspirin, Clopidogrel, Inguinal hernia, Inguinal hernia repair

## Abstract

**Introduction:**

There remains no consensus surrounding the safety of prescribing anti-platelet therapies (APT) prior to elective inguinal hernia repair (IHR).

**Aims:**

To perform a systematic review and meta-analysis evaluating the safety profile of APT use in patients indicated to undergo elective IHR.

**Methods:**

A systematic review was performed in accordance with PRISMA guidelines. Meta-analyses were performed using the Mantel–Haenszel method using the Review Manager version 5.4 software.

**Results:**

Five studies including outcomes in 344 patients were included. Of these, 65.4% had APT discontinued (225/344), and 34.6% had APT continued (119/344). The majority of included patients were male (94.1%, 288/344). When continuing or discontinuing APT, there was no significant difference in overall haemorrhage rates (odds ratio (OR): 1.86, 95% confidence interval (CI): 0.29–11.78, *P* = 0.130) and in sensitivity analysis using only RCT data (OR: 0.63, 95% CI: 0.03–12.41, *P* = 0.760). Furthermore, there was no significant difference in reoperation rates (OR: 6.27, 95% CI: 0.72–54.60, *P* = 0.590); however, a significant difference was observed for readmission rates (OR: 5.67, 95% CI: 1.33–24.12, *P* = 0.020) when APT was continued or stopped pre-operatively. There was no significant difference in the estimated blood loss, intra-operative time, transfusion of blood products, rates of complications, cerebrovascular accidents, myocardial infarctions, or mortality observed.

**Conclusion:**

This study illustrates the safety of continuing APT pre-operatively in patients undergoing elective IHR, with similar rates of haemorrhage, reoperation, and readmission observed. Clinical trials with larger patient recruitment will be required to fully establish the safety profile of prescribing APT in the pre-operative setting prior to elective IHR.

**Supplementary Information:**

The online version contains supplementary material available at 10.1007/s11845-023-03480-w.

## Introduction

Elective inguinal hernia repair (IHR) is among the most common operation performed worldwide [1]. As the surgical community is now cognisant of our ever-aging global population, candidates indicated to undergo IHR are likely to have more comorbidities and require more medications, including the post-event medical treatment of cerebrovascular accidents and acute coronary syndromes, all of which require lifelong anti-platelet therapy (APT) [2]. While it is well recognised that patients receiving APT are at risk of haemorrhage [3], these patients are also at an increased risk of clotting and thromboembolic events [4]. These risks are accentuated in the peri-operative setting, particularly when regularly administered APT are placed on hold to minimise intra-operative haemorrhage in complex patients with extensive cardiopulmonary comorbidity [4].

Current guidelines from societies such as the European Society of Cardiology recommend the cessation of clopidogrel 7 days prior to non-cardiac surgery, while aspirin therapy should be stopped 3 days prior [5, 6]. Notwithstanding these recommendations, there remains no specific consensus regarding the safety of continuing or discontinuing APT in patients undergoing elective IHR. Given the increased proportion of patients who are now receiving APT [7], coupled with the increased number of elective IHRs performed each year [1], it is imperative to assess the safety profile of stopping and continuing APT for those due to undergo elective IHR. Accordingly, the aim of the current study was to perform a systematic review and meta-analysis evaluating the safety profile of APT use in patients indicated to undergo elective IHR.

## Methods

### Materials and methods

A systematic review was performed in accordance with the preferred reporting items for systematic reviews and meta-analyses (PRISMA) checklist and meta-analysis and systematic reviews of observational studies (MOOSE) guidelines [8, 9]. This study was registered with the International Prospective Register of Systematic Reviews (PROSPERO – CRD42023388552). Local institutional ethical approval was not required for this study.

### Search strategy

An electronic search was performed of the PubMed, Embase, and Cochrane databases on the 31 December 2022 for relevant studies suitable for inclusion in this study. The search was performed of all fields under the following headings: (anti-platelet), (aspirin), (clopidogrel), and (inguinal hernia), which were linked with the Boolean operators ‘AND’ and ‘OR’. Included studies were limited to those published in the English language and to studies with full-text articles available. Included studies were not restricted based on the year of publication. Initially, all titles were screened, and studies deemed appropriate had their abstracts and full texts reviewed.

### Inclusion and exclusion criteria

Studies meeting the following inclusion criteria were included: (1) Studies assessing the impact of APT on surgical outcomes in patients undergoing elective IHR. Studies meeting any of the following exclusion criteria were excluded from this study: (1) Studies not assessing the impact of APT on surgical outcomes in patients undergoing elective IHR; (2) studies not reporting outcomes specific to IHR; (3) studies with patients on dual anti-platelet therapy (DAPT), (4) review articles; (5) studies including less than 10 patients in their series; or (6) editorial articles.

### Data extraction and quality assessment

Two independent reviewers performed the literature search using a predesigned search strategy. Duplicate studies were manually removed. Each reviewer then reviewed the titles, abstracts, and/or full texts of the retrieved manuscripts to ensure all inclusion criteria were met, before extracting the following data: (1) first author name; (2) year of publication; (3) study design and level of evidence; (4) country of origin; (5) number of patients who underwent IHR repair included; (6) number of patients who had APT continued prior to IHR; (7) number of patients who had APT stopped prior to IHR; (8) number of patients who were not receiving APT prior to IHR; (9) basic clinicopathological data (e.g. age at diagnosis, gender); and (10) post-operative surgical outcomes from each study. This included studies comparing patients who were previously prescribed APT pre-operatively who then had their therapy stopped or continued in the pre-operative setting. Methodological and risk of bias assessment of the included studies was undertaken using the Newcastle–Ottawa Risk of Bias Assessment tool for observational studies [10].

### Statistical analysis

Fisher’s exact (†) test was used as appropriate to determine the association between APT use and post-operative surgical outcomes [11]. Thereafter, post-operative surgical outcomes were expressed as dichotomous or binary outcomes, reported as odds ratios (OR) and 95% confidence intervals (95% CIs) following estimation using the Mantel–Haenszel method. Variables represented as continuous data were expressed as means with associated standard error (SE), before being utilised to calculate mean difference (MD) with associated 95% CI. Data specific to patient outcomes and APT use were directly extracted from tables and study text. Either fixed or random-effects modelling was applied on the basis of whether significant heterogeneity (*I*^*2*^ > 50%) existed between studies included in the analysis. Symmetry funnel plots were used to assess publication bias. Statistical heterogeneity was determined using *I*^*2*^ statistics. All tests of significance were two-tailed with *P* < 0.050 indicating statistical significance. Descriptive statistics were performed using the Statistical Package for Social Sciences (SPSS) version 26 (International Business Machines Corporation, Armonk, New York). Meta-analysis was performed using Review Manager (RevMan), Version 5.4 (Nordic Cochrane Centre, Copenhagen, Denmark).

## Results

### Literature search

**Fig. 1:**
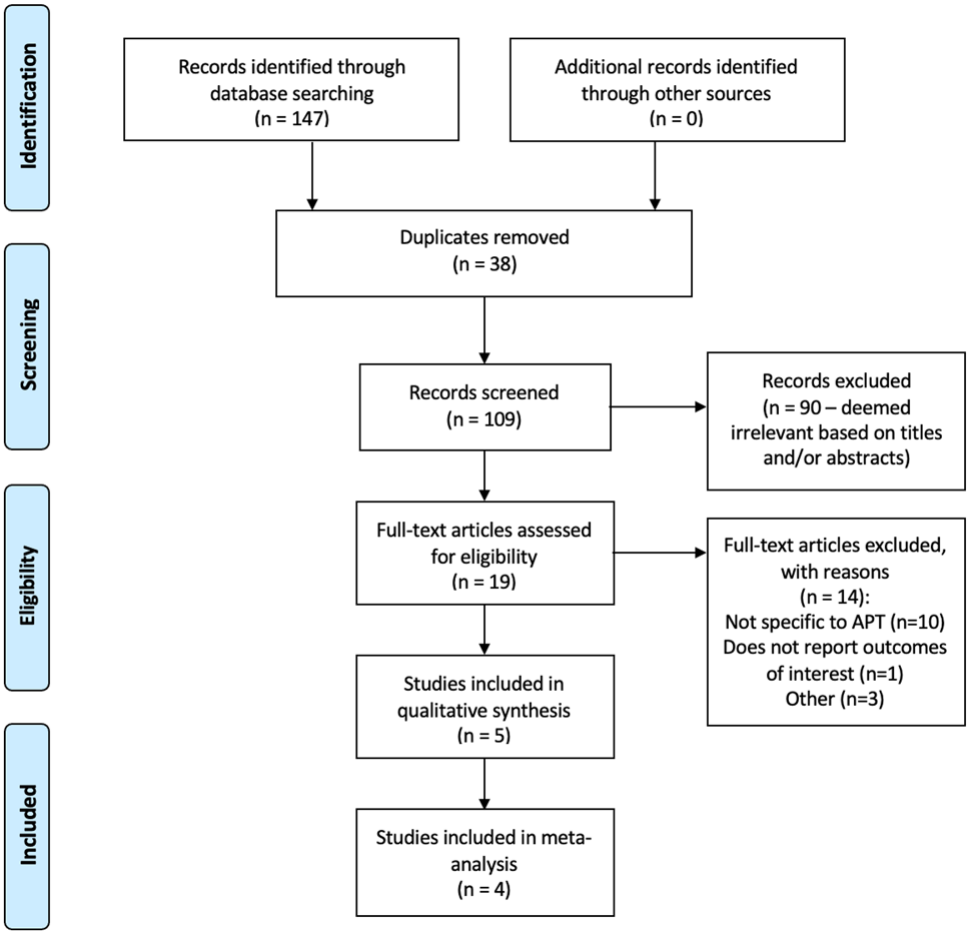
PRISMA flowchart illustrating the systematic search process

The systematic search strategy identified a total of 147 studies, of which 38 duplicate studies were manually removed. The remaining 109 studies had their titles screened for relevance before 23 abstracts, and 19 full texts were reviewed for edibility. In total, 5 studies fulfilled the inclusion criteria and were included in this systematic review [12–16]. Of these, 4 studies were eligible for inclusion in this meta-analysis [12–15] (Fig. 1).

### Study characteristics

Of the 5 included studies, 40.0% provided data from American [12, 16] and European [13, 14] translational research facilities respectively (both 2/5). There were 2 prospective, randomised clinical trials (RCTs) included in this study (40.0%, 2/5) [12, 13], and the remaining 3 studies were of retrospective design (60.0%, 3/5) [14–16]. Publication dates of included studies ranged from 2011 to 2016. Basic study data from the included 5 studies are outlined in Table 1. Risk of bias performed using the Newcastle–Ottawa Scale for observational studies is outlined in Table 1.

**Table 1:** Details from the five included studies

Author	Year	Country	LOE	Design	Therapy	Details regarding anti-platelet cessation	NOS
Antolovic	2012	Germany	I	RCT	Aspirin	Stopped less than 5 days versus stopped before 5 days	8
Chu	2016	USA	I	RCT	Clopidogrel	Stopped before 7 days versus continued until day of surgery	8
Chu	2011	USA	III	RC	Clopidogrel	Stopped before 7 days versus continued within 7 days of surgery	6
Mogrampi	2016	Greece	III	RC	Anti-platelets	Anti-platelet therapy was not discontinued	6
Ong	2016	Singapore	III	RC	Aspirin	Stopped before 3–7 days versus continued until day of surgery	6

*LOE* level of evidence, *USA* United States of America, *RCT* randomised control trial, *RC* retrospective cohort

### Patient demographics

In total, 5 studies reported outcomes in 344 patients who either had their APT stopped or continued in the pre-operative setting prior to IHR [12–16]. Of these, 65.4% had their APT continued (225/344) and 34.6% had their APT continued (119/344). Three studies reported patient gender [14–16], and the majority of patients were male (94.1%, 288/344). All 5 included studies reported patient age [12–16], and the mean age of included patients was 67 years (range: 22–88 years) (Table 2). There was a non-significant difference in the American Society of Anesthesiologists (ASA) grade for both groups (*P* = 0.055, †).

**Table 2:** Patient demographics

Author	Year	Number	*N* cont	*N* stop	Males	Females	Mean age (range)
Antolovic	2012	23	12	11	-	-	68 years (22–88)
Chu	2016	15	9	6	-	-	68 years
Chu	2011	46	26	20	39	7	74 years
Mogrampi	2016	118	118	-	109	9	56 years
Ong	2016	142	60	82	140	2	70 years
	-	344	225	119	288	18	67 years (22–88)

*N* number, *cont.* continued anti-platelet therapy pre-operatively, *stop* stopped anti-platelet therapy pre-operatively

### Haemorrhage

**Fig. 2:**
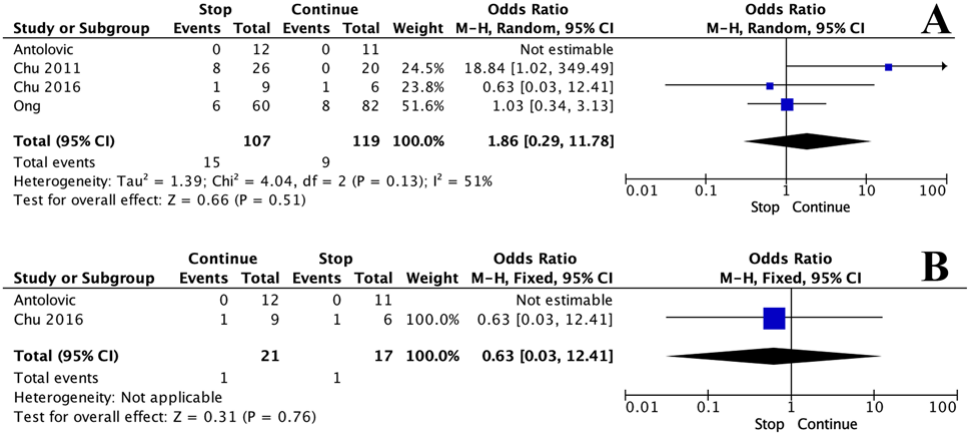
Forest plots illustrating the risk of haemorrhage when stopping and continuing for the **A** overall cohort and **B** using randomised control trial data only

There was no significant difference in haemorrhage rates when APT was continued or stopped pre-operatively [4.9% (17/225) vs. 7.6% (9/119), *P* = 1.000, †]. At meta-analysis, there was no significant difference in haemorrhage rates when APT was continued or stopped pre-operatively (OR: 1.86, 95% CI: 0.29–11.78, *P* = 0.130, *I*^*2*^ = 51%) (Fig. 2A). Furthermore, when performing a sensitivity analysis using RCT data only, there was no significant difference in haemorrhage rates (OR: 0.63, 95% CI: 0.03–12.41, *P* = 0.760) (Fig. 2B).

### Reoperation rates

**Fig. 3:**
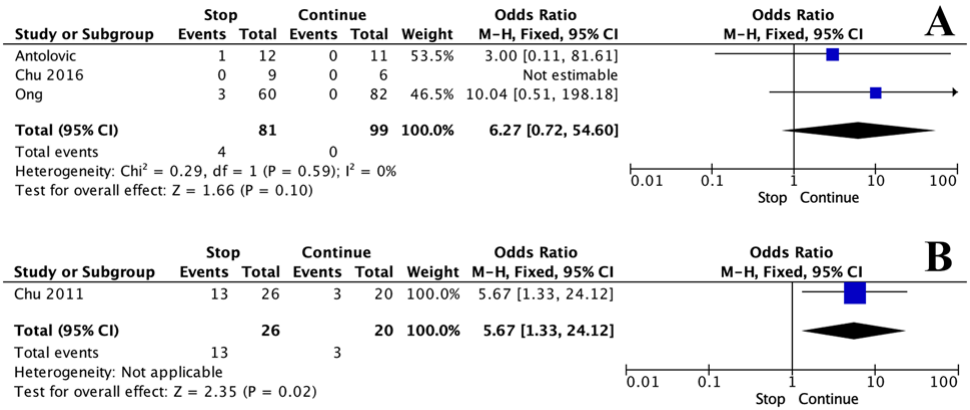
Forest plots illustrating the **A** reoperation and **B** readmission rates for those who had anti-platelet therapies stopped and continued prior to elective inguinal hernia repair

There was no significant difference in reoperation rates when APT was continued or stopped pre-operatively [4.9% (4/81) vs. 0.0% (0/99), *P* = 0.175, †]. At meta-analysis, there was no significant difference in reoperation rates when APT was continued or stopped pre-operatively (OR: 6.27, 95% CI: 0.72–54.60, *P* = 0.590), *I*^*2*^ = 0%) (Fig. 3A).

### Readmission rates

Although only reported in the study by Chu et al. [14], there was a significant difference observed in readmission rates when APT was continued pre-operatively [50.0% (13/26) vs. 15.0% (3/12), *P* = 0.027, †]. At meta-analysis, there was a significant difference observed in readmission rates when APT was continued pre-operatively (OR: 5.67, 95% CI: 1.33–24.12, *P* = 0.020) (Fig. 3B).

### Intra-operative outcomes

**Fig. 4:**
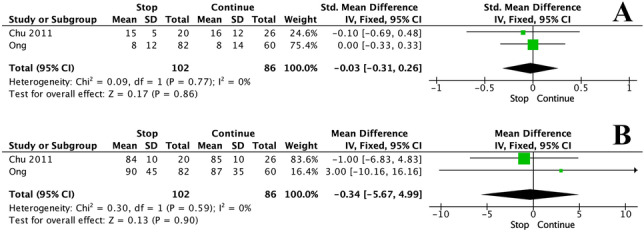
Forest plots illustrating the **A** estimated blood loss and **B** intra-operative time for those who had anti-platelet therapies stopped and continued prior to elective inguinal hernia repair

At meta-analysis, there was no significant difference in estimated blood loss (EBL) (MD: − 0.03, 95% CI: − 0.31–0.26 *P* = 0.860, *I*^*2*^ = 0%) (Fig. 4A) or in intra-operative time (IOT) (MD: − 0.34, 95% CI: − 5.67–4.99 *P* = 0.900, *I*^*2*^ = 0%) (Fig. 4B) when APT was continued or stopped pre-operatively. Symmetry funnel plots to assess bias are outlined in detail in the Supplementary Material.

### Other outcome measures

There was no significant difference observed in complication rates when APT was continued or stopped pre-operatively [1.2% (2/165) vs. 0.0% (0/37), *P* = 0.465, †]. None of the patients included in this study required a transfusion of blood products, suffered a cerebrovascular accident, myocardial infarction, or mortality in this study, limiting the analysis which could be performed surrounding these outcome measures.

## Discussion

The most important findings in this systematic review and meta-analysis are the data illustrating a non-significant difference in post-operative haemorrhage and reoperation rates observed irrespective of APT being stopped or continued pre-operatively in patients undergoing elective IHR. These results accentuate the safety profile of continuing APT in the peri-operative setting surrounding elective IHR, despite relying on absolute differences from crude data illustrating increased haemorrhage and reoperation rates in those who continue APT use pre-operatively. Furthermore, a sensitivity analysis performed using just RCT data was performed to assess the impact of APT on post-operative haemorrhage, further validating these results. Thus, this data highlights the safety profile of continuing APT pre-operatively, with limited premise to discontinue such therapies pre-operatively, unless in the settings of complex cases under the clear direction of physicians with expertise in coagulation and haematological medicine.

Interestingly, patients who had APT continued prior to elective IHR were more likely to be readmitted following discharge from hospital following surgery. This is an unanticipated finding, and one which may be scrutinised when a thorough assessment of the data supporting this finding is performed: Of note, Chu et al. were the sole study reporting readmission rates following the continuation and discontinuation of APT pre-operatively [14], with outcomes favouring stopping APT pre-operatively in order to prevent readmissions [continued APT: 50.0% (13/26) vs. discontinued APT: 15.0% (3/20)]. Importantly, 8 of the 13 patients were readmitted for haemorrhage (61.5%), and it is imperative to highlight that Chu et al. included a total of 46 patients in their study. Therefore, it is likely that this study is underpowered to provide coherent outcomes in relation to this outcome measure. While these data cast doubt into the safety of using APT in the peri-operative setting for those undergoing IHR, it is imperative to evaluate the more robust data reported for stronger outcome measures, such as haemorrhage (*n* = 344) and reoperation rates (*n* = 180), respectively. Thus, the next generation of prospective studies may utilise readmission rates post APT in the setting of elective IHR as a secondary outcome measure to fully establish the long-term safety of APT following discharge from the acute hospital setting.

This is not the first systematic review performed assessing the safety profile of anti-platelet and anti-coagulation therapies in the settings of patients due to undergo elective IHR. Li et al. performed a systematic review of 13 studies to determine consensus surrounding blood thinning medications in the peri-operative setting and similarly to the results if the current meta-analysis [17], concluded that ‘there is no need to stop anti-platelet therapy (Aspirin or Clopidogrel)’. In addition, these authors recommended the tailoring of anti-coagulation and warfarin prescription in a case-by-case basis, due to the heterogeneity of indications and complexity of each patient’s conditions when in receipt of such therapies. Importantly, the current analysis supports the consensus of these previous authors, while providing accurate ‘real world’ data illustrating the risk of APT use in this setting. In addition, the data presented in the current study may be of use to the surgeon to aid pre-operative patient counselling surrounding the safety profile of continuing APT prior to elective IHR.

The current study suffers from several innate limitations. Firstly, this analysis included data from just 344 patients limiting the robustness of conclusions which may be drawn from this study. Moreover, with the inclusion of more available studies, it is possible the difference observed in outcome measures may be accentuated and potentially may facilitate significant differences among outcome measures (i.e. haemorrhage and reoperation rates). Secondly, and similarly, there were no reported events of transfusion of blood products, cerebrovascular accident, myocardial infarction, or mortality in this study, which again is potentially due to type II statistical errors observed across the included studies. Thirdly, the management paradigm of IHR has evolved considerably in recent decades with the advent of minimally invasive approaches (i.e. total extraperitoneal and transabdominal approaches), yet the current analysis falls short of evaluating differences in outcomes for such approaches to IHR. Despite these limitations, the authors wish to highlight this study provides high-quality evidence supporting the safety of continuing APT prior to elective IHR.

In conclusion, this systematic review and meta-analysis illustrates the safety of continuing APT pre-operatively in patients undergoing elective IHR. This study illustrated a non-significant difference in post-operative haemorrhage and reoperation rates observed irrespective as to whether APT was stopped or continued pre-operatively in patients undergoing elective IHR. The provision of clinical trials with larger patient recruitment will be necessitated in order to fully establish the safety profile of prescribing APT in the pre-operative setting prior to elective IHR. Until then, a case-by-case approach in relation to the use of APT in the pre-operative setting prior to IHR will be at the discretion of the surgeon and anaesthetic team who are responsible for the patients care.

### Supplementary Information

Below is the link to the electronic supplementary material.

Supplementary file1 (DOCX 345 KB)
