# Supplementary material for: Evaluating the safety profile of anti-platelet therapy in patients undergoing elective inguinal hernia repair: a systematic review and meta-analysis
Source: Ir J Med Sci. 2023 Aug 1;193(2):897–902. doi: 10.1007/s11845-023-03480-w (PMC10961273; doi:10.1007/s11845-023-03480-w)
Supplement: Supplementary file 1 — Supplementary file1 (DOCX 345 KB) [file 11845_2023_3480_MOESM1_ESM.docx]

**Evaluating the safety profile of antiplatelet therapy in patients undergoing elective inguinal hernia repair – A systematic review and meta-analysis**

Matthew G. Davey MCh MRCSI PhD^1^, William P. Joyce MCh FRCSEd, FACS, FRCS^1,2^

^1^Royal College of Surgeons Ireland, 123 St. Stephens Green, Dublin 2, D02 YN77

^2^Department of Surgery, Galway Clinic, Co. Galway H91 HHT0

Supplementary Material

Symmetry funnel plots to assess bias for (A) haemorrhage, (B) haemorrhage using only randomised clinical trial data, (C) reoperation, (D) readmission, (E) intraoperative estimated blood loss, and (F) intraoperative time.

A


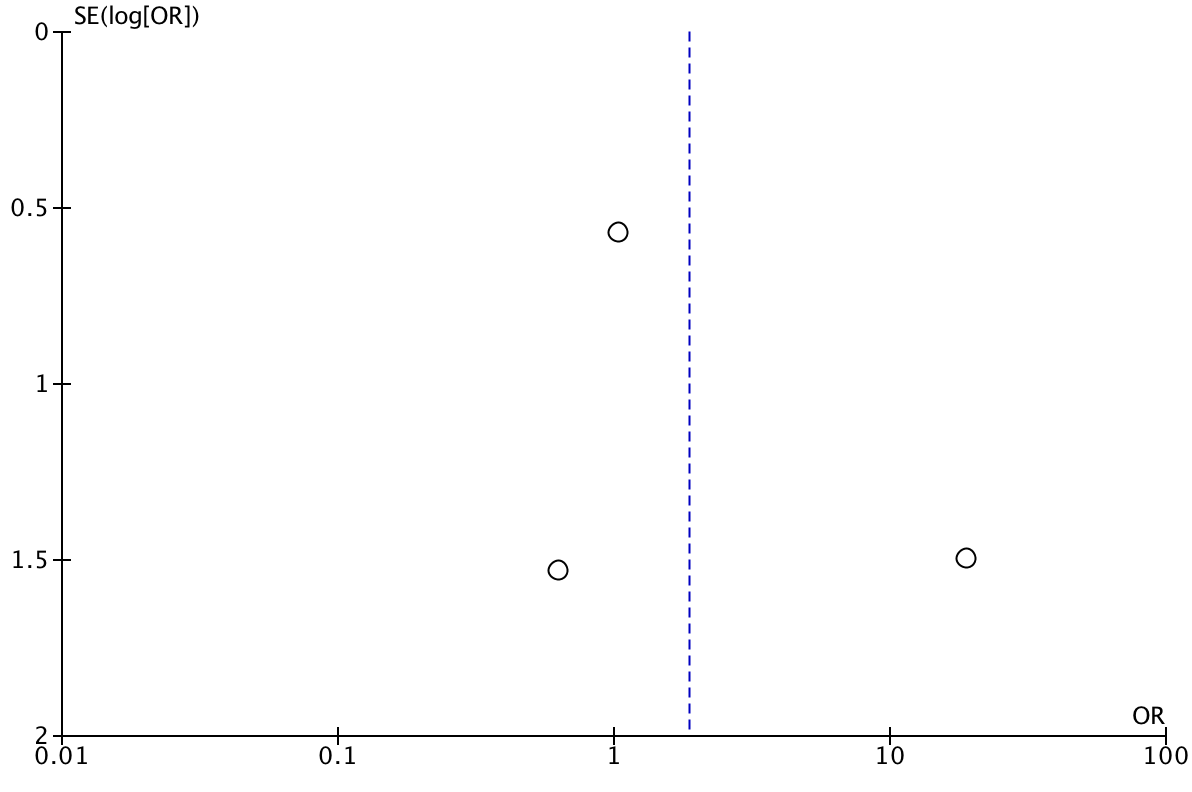


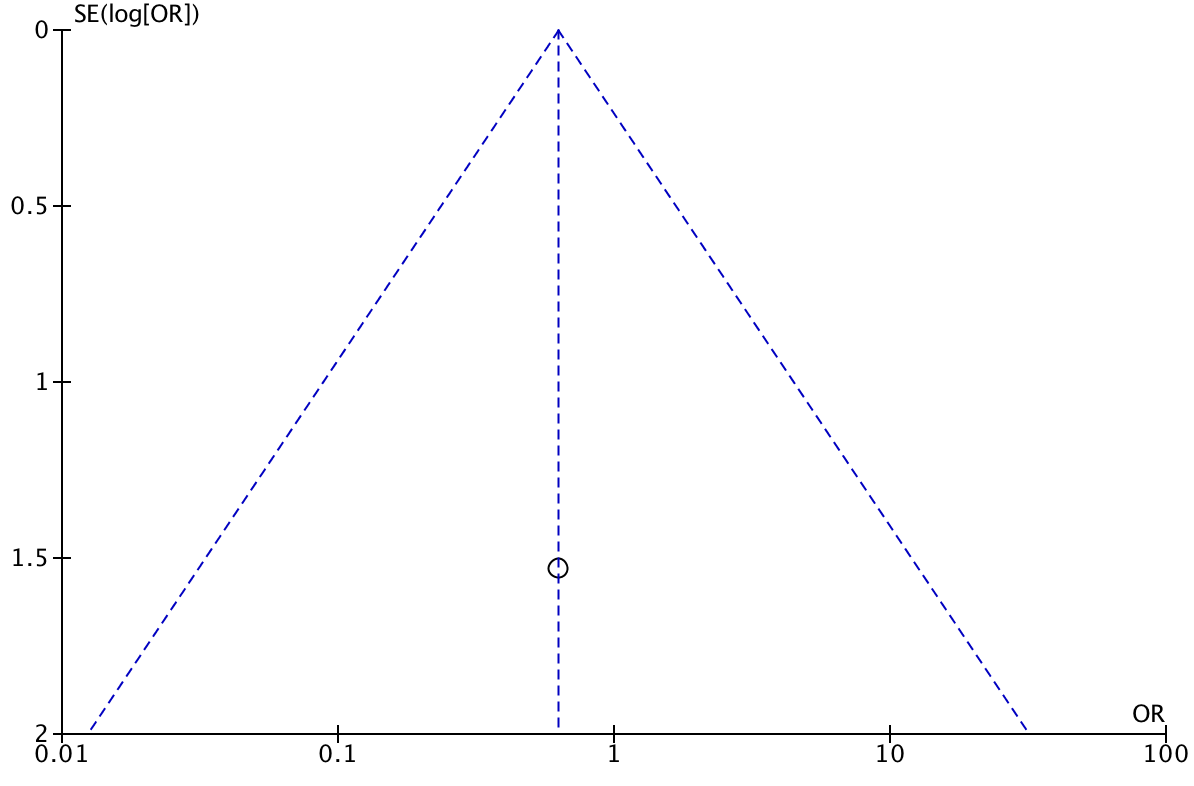


B


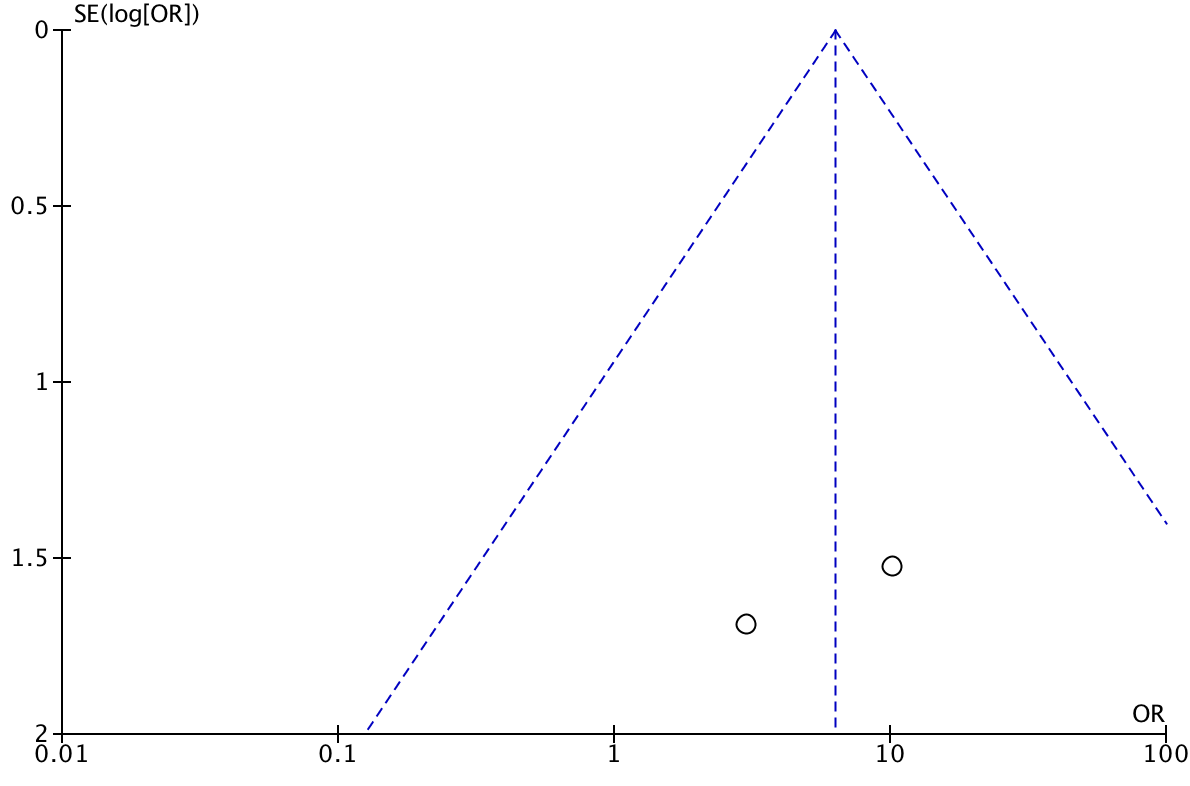


C


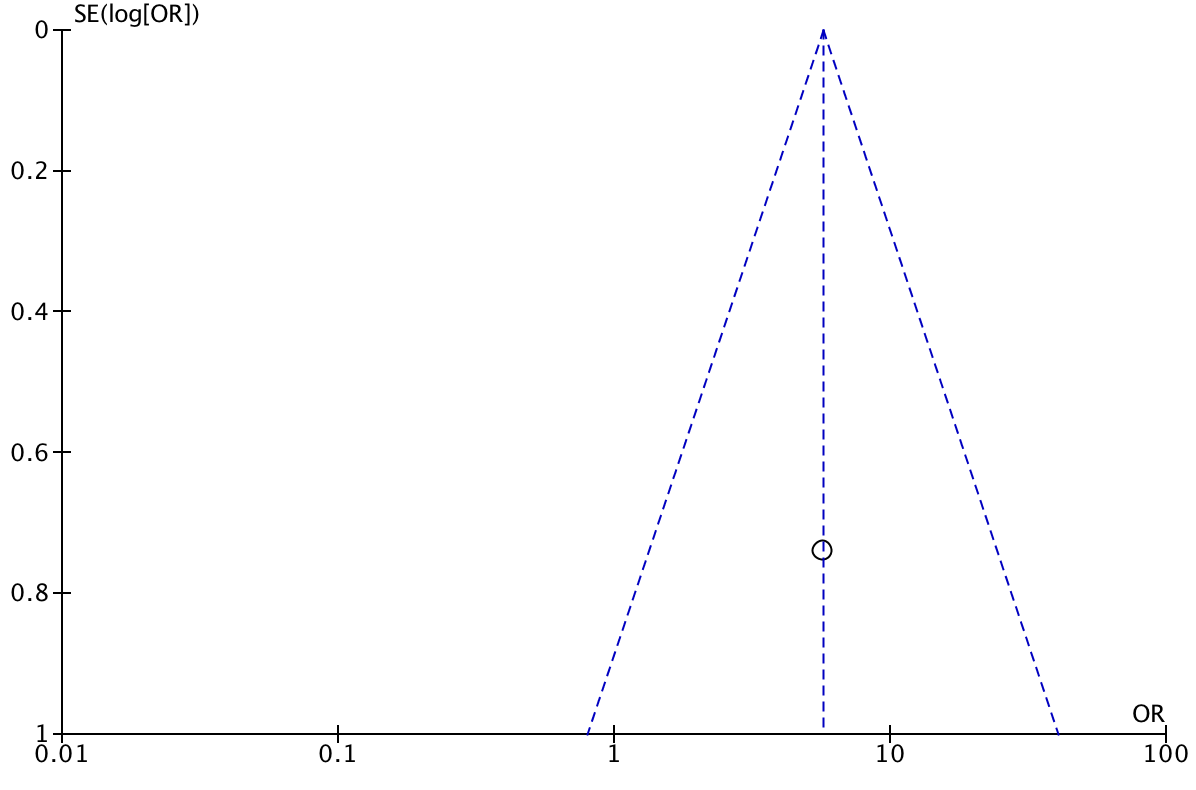


D


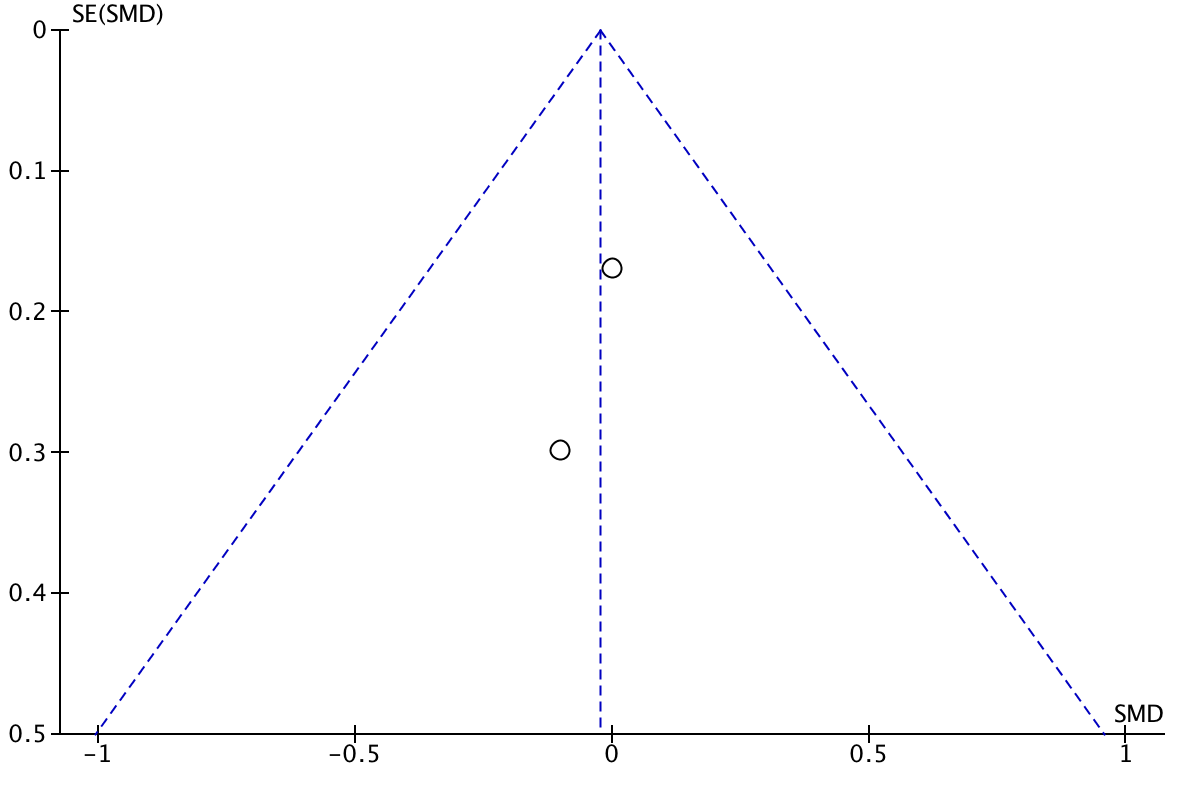


E


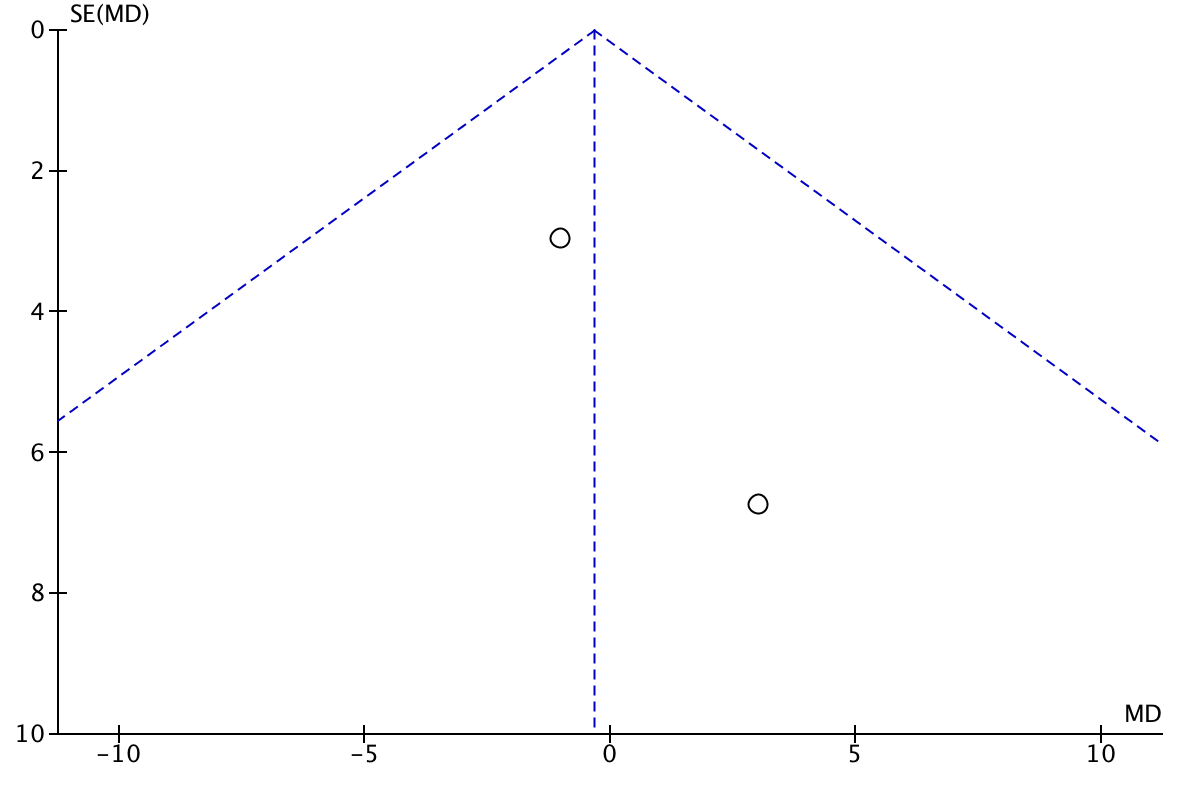


F
